# Botanicals in Postmenopausal Osteoporosis

**DOI:** 10.3390/nu13051609

**Published:** 2021-05-11

**Authors:** Wojciech Słupski, Paulina Jawień, Beata Nowak

**Affiliations:** Department of Pharmacology, Wroclaw Medical University, ul. J. Mikulicza-Radeckiego 2, 50-345 Wrocław, Poland; wojciech.slupski@umed.wroc.pl (W.S.); paulina.jawien@umed.wroc.pl (P.J.)

**Keywords:** osteoporosis, menopause, botanicals, herbs

## Abstract

Osteoporosis is a systemic bone disease characterized by reduced bone mass and the deterioration of bone microarchitecture leading to bone fragility and an increased risk of fractures. Conventional anti-osteoporotic pharmaceutics are effective in the treatment and prophylaxis of osteoporosis, however they are associated with various side effects that push many women into seeking botanicals as an alternative therapy. Traditional folk medicine is a rich source of bioactive compounds waiting for discovery and investigation that might be used in those patients, and therefore botanicals have recently received increasing attention. The aim of this review of literature is to present the comprehensive information about plant-derived compounds that might be used to maintain bone health in perimenopausal and postmenopausal females.

## 1. Introduction

Women’s health and quality of life is modulated and affected strongly by hormone status. An oestrogen level that changes dramatically throughout life determines the development of women’s age-associated diseases. Age-associated hormonal imbalance and oestrogen deficiency are involved in the pathogenesis of various diseases, e.g., obesity, autoimmune disease and osteoporosis. Many female patients look for natural biological products deeply rooted in folk medicine as an alternative to conventional pharmaceutics used as the prophylaxis of perimenopausal health disturbances. This review will focus on botanicals and plant derived substances that may be used to maintain bone health in perimenopausal and postmenopausal females.

Osteoporosis is a systemic bone disease characterized by the reduced bone mass and deterioration of bone microarchitecture leading to bone fragility and the increased risk of fractures [1]. Osteoporosis-associated fragility fractures constitute a major health problem all over the world. It is estimated that more than 40 million American citizens over 50 years of age are at risk of osteoporotic fractures, and that due to the demographic changes, this number will at least double until the year 2040 [2]. It is also predicted that 25% of people over 50 who have experienced osteoporotic hip fracture will die within a year [2]. Hypogonadism, mainly associated with menopause, is the main cause of osteoporosis. High social and individual costs of osteoporosis and its complications remain a challenge for health systems, especially because most of the patients with osteoporosis remain untreated. The data indicate that almost 60% patients at high risk of osteoporotic fractures are not receiving osteoprotective treatment [3]. Additionally, a decrease in the usage of antiosteoporotic drugs, especially bisphosphonates, has been observed in recent years [3]. Oral bisphosphonates, that bind to hydroxyapatite and inhibit osteoclastic bone resorption, are the drug of choice for the treatment of primary osteoporosis. However, they are associated with side effects including oesophagitis and oesophageal ulcers, jaw osteonecrosis, and atypical femoral fractions. In case of intolerance or lack of efficacy, they might be switched to intravenous bisphosphonates, strontium ranelate, denosumab, teriparatide, abaloparatide or romosozumab. As additional options in postmenopausal women, raloxifene and hormonal replacement therapy may be used [4]. However, as those pharmaceutics are associated with various side effects, many women seek for botanicals as an alternative therapy.

**Figure 1:**
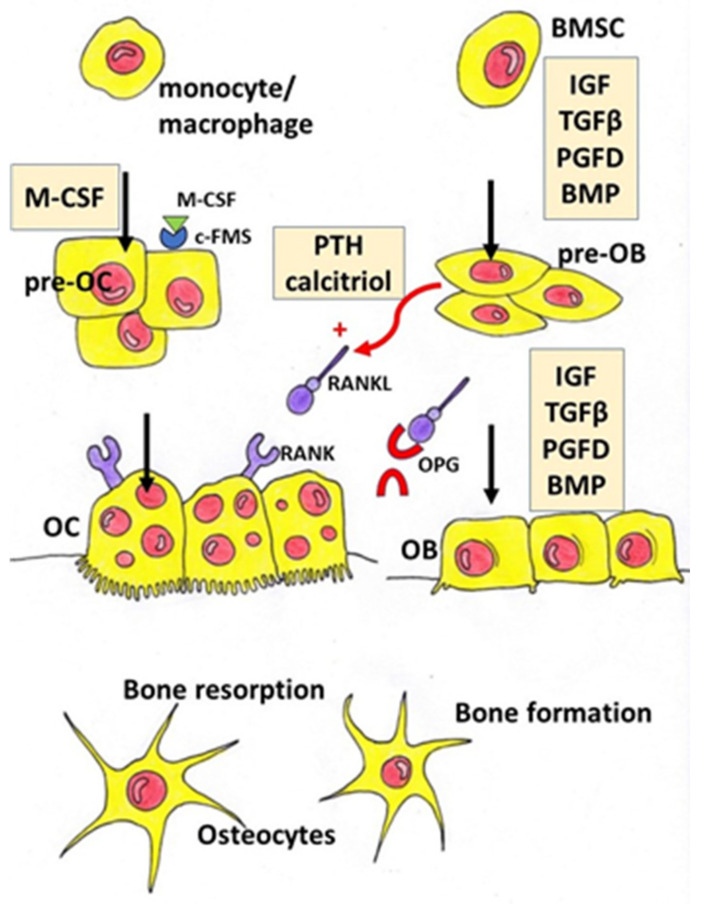
Schematic diagram representing regulation of osteoblast and osteoclast differentiation. BMP—bone morphogenic protein, BMSC—bone marrow-derived mesenchymal stem cells, c-FMS—colony-stimulating factor-1 receptor, IGF—insulin-like growth factor, M-CSF—macrophage colony-stimulating factor, OB—osteoblast, OC—osteoclast, OPG—osteoprotegerin, PGFD—platelet-derived growth factor, pre-OB—pre-osteoblasts, pre-OC—pre-osteoclasts, PTH—parathyroid hormone, RANK—Receptor Activator for Nuclear Factor κB, RANKL—Receptor Activator for Nuclear Factor κB Ligand, TGFβ—tumour growth factor β.

Bones undergo continuous remodelling, osteoblasts synthetize the bone matrix and, at the same time, osteoclasts degrade bone tissue. In physiological conditions, we observe the balance between the resorption and formation of bone tissue. This balance depends on the activity, differentiation, and apoptosis of bone forming osteoblasts and bone-resorbing osteoclasts. Multiple factors and signalling pathways modulate bone homeostasis (Figure 1). Bone cells’ activity is controlled, among others, by growth factors (IGF—insulin-like growth factor, TGFβ—tumour growth factor β, PDGF—platelet-derived growth factor), bone morphogenic proteins (BMPs), hormones (parathormone, thyroid hormones, sex hormones, insulin, prolactin, growth hormone) and vitamins (vitamin D). Wnt, BMPs and TGFβ pathways interact with other signalling molecules such as basic fibroblast growth factor (bFGF), Hedgehog (Hh) and IGF-1, and regulate the differentiation and activity of osteoclasts [5]. Runx2 (Runt-related transcription factor 2) and OSX (Osterix) are the main transcription factors involved in the modulation of osteoblast differentiation. Osteoclastogenesis is regulated by two main pathways: RANK/RANKL (Receptor Activator for Nuclear Factor κB/Receptor Activator for Nuclear Factor κB Ligand) and M-CSF/c-FMS (the macrophage colony-stimulating factor/colony-stimulating factor-1 receptor) system. Parathyroid hormone (PTH) and calcitriol induce RANKL synthesis in osteoblasts and afterwards promote osteoclastogenesis through RANK activation. RANK activation is counteracted by OPG (osteoprotegerin), which is a decoy receptor of free RANKL. M-CSF/c-FMS interaction leads to mitogen-activated protein kinase (MAPK) activation that induces RANKL production and activates AKT/mTOR (protein kinase B/mechanistic target of rapamycin) pathway engaged in the survival of osteoclasts [5].

**Figure 2:**
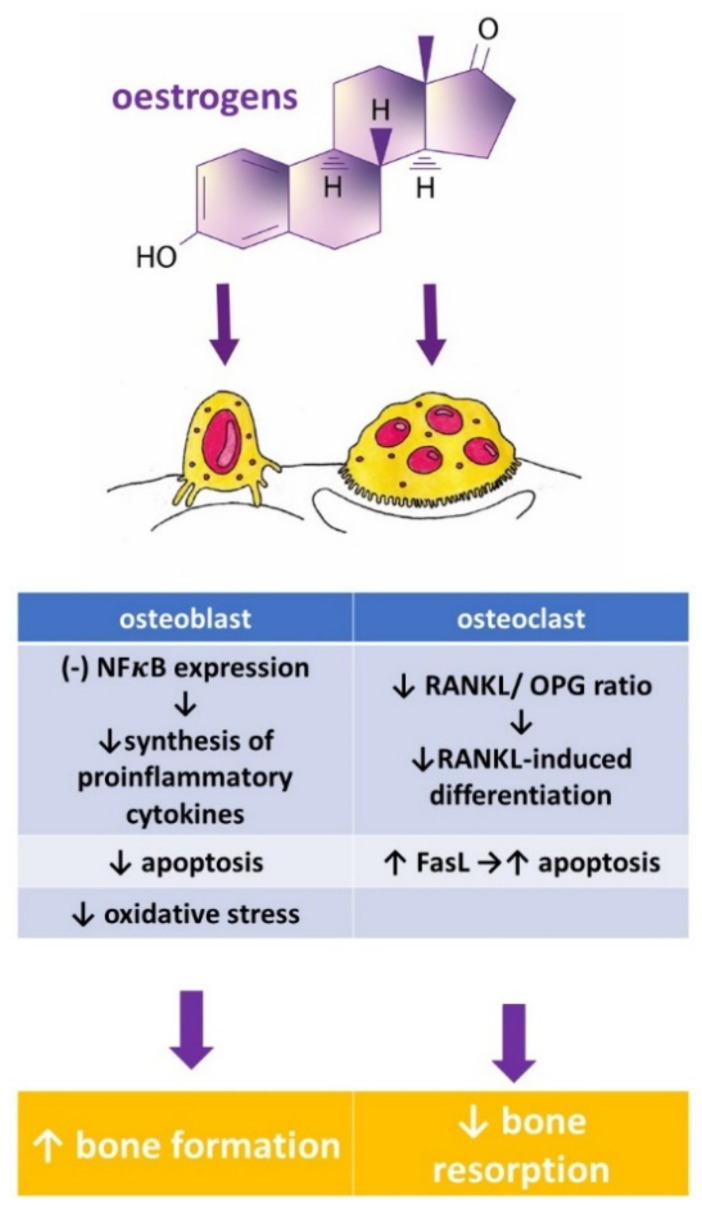
Influence of oestrogens on osteoblast and osteoclast function, and bone turnover. FasL—Fas Ligand, NFκB—Nuclear Factor κB, OPG—osteoprotegerin, RANKL—Receptor Activator for Nuclear Factor κB Ligand, ↑—increased, ↓—decreased

Oestrogen plays an important role in maintaining bone mineral density in both rodents and humans (Figure 2). A decrease in the oestrogen level associated with menopause leads to a decrease in bone mineral density (BMD) that increases the risk of fractures [6,7]. The protective effect of oestrogen in bone is due to many mechanisms. Oestrogen, among other things, inhibits bone resorption by the suppression of the synthesis of proinflammatory cytokines in osteoblasts via the inhibition of nuclear factor-kappa B (NFκB) signalling pathway [8]. They also activate the transcription of a gene encoding Fas Ligand (FasL) in osteoblasts. Soluble FasL (sFasL) released from the osteoblast binds to the transmembrane Fas receptor (FasR) on the osteoclast’s surface and induces the apoptosis of osteoclasts [9]. Additionally, oestrogen decreases the RANKL/OPG ratio and prevents bone resorption [10].

Women’s health and quality of life are modulated and affected strongly by hormone status. An oestrogen level that changes dramatically determines the development of women’s age-associated diseases. Age-associated hormonal imbalance and oestrogen deficiency are involved in the pathogenesis of various diseases, e.g., obesity, autoimmune diseases, and osteoporosis. As postmenopausal osteoporosis is characterised by bone resorption that exceeds bone formation, antiresorptive drugs are one of the therapeutic options and most current therapies exert mainly antiresorptive effects. Another therapeutic solution may be the use of anabolic drugs that would enhance bone formation. Bone morphogenic protein (BMP), Wnt, and insulin-like growth factor 1 (IGF1) are the key molecules involved in the regulation of osteoblast formation and activation [11,12,13]. Oestrogens, SERMs (selective oestrogen receptor modulators), bisphosphonates, strontium ranelate, denosumab, teriparatide, abaloparatide or romosozumab are clinically used as effective therapies against postmenopausal osteoporosis [4]; however, their usage is associated with the established risk of the side effect. Therefore, many female patients look for natural biological products deeply rooted in folk medicine as an alternative to conventional pharmaceutics used as the prophylaxis of perimenopausal health disturbances. This review will focus on botanicals and plant-derived substances that may be used to maintain bone health in perimenopausal and postmenopausal females. The aim of the review is to present the currently available results of clinical and preclinical studies, investigating the influence of plant-derived extracts and compounds on menopause-associated disturbances of bone metabolism. For the purpose of the article, we defined botanicals as substances obtained or derived from plants, such as a plant part or the extract, or compounds isolated from plants or their extracts. While searching for the information in PubMed and Google Scholar, we tried not to limit our research to Chinese traditional medicine, but to broaden it by including less known European plants, e.g., *Humulus lupulus* L. or *Equisetum arvense* L. We focused on the research reported after 2010, but we did not exclude earlier studies in our review. Table 1 summarizes the information about the main active ingredients discussed in the article, and Table 2 clinical studies and their main findings.

## 2. Phytoestrogens

Phytoestrogens are naturally occurring nonsteroidal plant compounds that resemble oestrogens and have oestrogenic and/or antiestrogenic activity. They can be divided into two main groups: flavonoids and non-flavonoids. Isoflavones, coumestans, and prenylflavonoids belong to flavonoids, and lignans belong to non-flavonoids [62].

### 2.1. Isoflavones

Isoflavones are phenolic compounds that belong to the most estrogenic plant-derived substances. Their chemical structure is similar to that of oestradiol. They include, among others, genistein, daidzein, glycitein, biochanin A, and formononetin (Table 3). The main source of isoflavones are legumes belonging to *Fabaceae*: soybean (*Glycine max*) as a source of genistein, daidzein, and glycitein, and red clover (*Trifolium pratense*) as a source of biochanin A and formononetin [62]. In the group of plants containing isoflavones, there are also alfalfa (*Medicago saltiva* L.), beans (green bean, mung bean), psoralea (*Psoralea corylifolia*) and *kudzu* root (*Pueraria lobata* L.) [14]. In the human gastrointestinal tract formononetin, contained in dietary supplements based on red clover, is transformed to daidzein [63]. The amount of isoflavones in soybeans ranges from 1.2 to 4.2 mg per g of dry weight, whereas in red clover, it ranges from 10 to 25 mg per g of dry weight [14]. Isoflavones exert the biologic effect due to two different mechanisms. On the one hand, they act through the classical oestrogen receptor (ER)-mediated signalling pathway, but additionally, it has been described that they may activate intracellular pathways such as protein tyrosine kinase, phospholipase C and MAPK [14]. As most isoflavones are ERβ-selective ligands, it can be supposed that they selectively target bone cells without having an undesired influence on other oestrogen-sensitive tissues, such as the breast and the uterus.

#### 2.1.1. Soybean in Clinical Trials

The soybean (*Glycine max* L.) is an annual plant belonging to the Fabaceae family, which grows mainly in Southwest Asia. It is a rich source of proteins and flavonoids, such as genistein, daidzein, biochanin A, and glycitein [64]. In soybean, the aglycones and conjugate forms of genistein account for 60% of isoflavones and daidzein for up to 30% [65].

Epidemiological studies have shown that the consumption of food that contains soy may reduce the risk of fracture in postmenopausal women, particularly among those in the early years following menopause [66]. Authors of several observational studies have noticed that populations with a high intake of soy are characterized with a lower incidence of osteoporotic fractures than Western populations [67,68].

To date, many clinical trials (Table 2), systematic reviews, and meta-analyses have been carried out on this topic. Their results suggest that soy phytoestrogens exert significant effects on bone metabolism, and that they inhibit, to some degree, osteoporosis in postmenopausal women [64]. In a study by Scheiber et al., administration of soy isoflavone (60 mg/day) during 12 consecutive weeks increased serum levels of phytoestrogens and ameliorated several key clinical risk factors for osteoporosis in healthy postmenopausal women [27]. Chiechi et al. have showed that supplementation with soya isoflavone in 2 meals twice a week increased bone osteoblastic activity and the serum osteocalcin level [28]. The 12-month-long administration of soy isoflavones (40 mg or 80 mg) maintained hip bone mineral content in later menopause or those with lower body weight or calcium [29]. In another clinical trial, it was reported that isoflavones (126 mg for 6 months) reduced bone loss [30]. The authors suggested that the observed beneficial effect was due to the inhibition of bone resorption in non-obese postmenopausal Chinese women. Similar findings were reported by Wu et al. after supplementation of early postmenopausal women with 75 mg of isoflavone conjugates/day for 1 year [31]. Increased volumetric bone mineral density (vBMD) in postmenopausal women was observed after taking a tablet with isoflavones 80 or 120 mg/day for 3 years [32]. An increase in serum concentrations of bone-specific alkaline phosphatase (BALP) and osteocalcin as markers of increased bone formation were caused by soya isoflavone supplementation (70 mg/day for 12 weeks) in Korean postmenopausal women [33]. Tit et al. reported the similar efficacy of hormonal replacement therapy (HRT) and phytoestrogens in terms of the effects on BMD and bone resorption in postmenopausal women. Two capsules with 40% standardized extract (20 mg soy isoflavones genistein and daidzein per capsule) given orally for 1 year significantly reduced bone resorption [34]. In a randomized clinical trial (RCT) with women during early menopause, Sathyapalan et al. compared the administration of 15 g soy protein with 66 mg isoflavone or 15 g soy protein alone. Moreover, a 6-month long observation revealed that soy reduced bone turnover markers, i.e., type I collagen crosslinked beta C-telopeptide (CTX, bone resorption marker) and type I procollagen-N-propeptide (P1NP, bone formation marker) [35]. However, the results are not consistent, with the study of Levis et al. reporting that supplementation with 200 mg of soy isoflavones daily for 2 years did not protect menopausal women against bone loss [36]. Kreijkamp-Kaspers et al. obtained convergent results—BMD did not differ significantly after the 99 mg supplementation of isoflavones in 25.6 g of soy protein for one year in postmenopausal women [37]. Similarly, another study indicated that the 110 mg/day of soy isoflavone aglycone given for one year in postmenopausal women did not prevent postmenopausal bone loss or affected bone turnover [38].

Phytoestrogen genistein given in the dose 54 mg daily for 1–3 years had positive effects on bone formation and osteopenia in postmenopausal women in several clinical trials [39,40,41,69]. According to a randomized, placebo-controlled, double-blind study reported by Lappe et al., a lower dose of genistein administered for a shorter time (30 mg daily for 6 months) also prevented osteoporosis development and reduced fracture risk in postmenopausal women [42]. Pawlowski et al. showed that isoflavones mixed in their natural ratios were more effective than genistein-rich soy supplement as bone-preserving agents in postmenopausal women treated for 50 days [70]. Moreover, genistein aglycone in tablets (54 mg daily for 2 years) exerted beneficial effects, not only in postmenopausal osteopenia, but also in women with osteoporosis [43].

The studies mentioned above show that isoflavones ameliorate menopause associated imbalance in bone turnover, protecting BMD and bone strength. These findings suggest that soybean phytoestrogens could be used as a dietary supplement to prevent postmenopausal osteoporosis. Meta-analysis of 63 RCTs found that genistein (54 mg/day) and ipriflavone (600 mg/day) in particular, have beneficial effects on BMD outcomes and are safe in postmenopausal women. Therefore, they may be considered as a complementary or alternative therapy and the prophylaxis of menopause-related osteoporosis [15]. Another meta-analysis of 26 randomized controlled trials (2652 oestrogen-deficient women) found that isoflavones attenuated moderately menopause-associated bone loss in the lumbar spine, femoral neck and distal radius [71]. Additionally, the authors noted that the effect of isoflavones on bone was greater if they were administered as aglycons. The protective influence of soy isoflavones (40–300 mg/day) on osteoporosis-related bone loss and bone mineral density in the femur, neck, lumbar spine and hip was also found in the meta-analysis of 52 controlled trials (5313 patients) [72]. However, the effectiveness of soy isoflavone supplementation in treatment and prophylaxis of osteoporosis in peri- and postmenopausal females remain debatable. In a systematic review of nine studies (1379 women), Perna et al. found no consensus regarding the protective effect of soy isoflavones (20–80 mg/day) on bone loss. However, the authors did not exclude the possible protective effect of soy isoflavones on bone metabolism. Similar conclusions of a systematic review of 23 clinical trials were reported by other authors that found only a minimal effect of isoflavones on bone mineral density in menopausal women [73]. Several other meta-analyses reported that the effects were minimal [43,74,75] or none, as mentioned above [36]. The antiosteoporotic effects of flavonoids seem to depend on the balance between their estrogenic agonist and antagonist properties [76]. Their beneficial influence on bone metabolism may also be derived from their other biochemical properties, including enzymatic inhibition of certain protein kinases or activation of estrogen type I receptors [64]. Some authors indicate that equol—an isoflavandiol produced by gut microflora from daidzein and possessing a higher estrogenic activity than the predominant flavonoids—may be responsible for the clinical effectiveness of flavonoids [77]. The discrepancies between the results of the reported studies may also be attributed to differences in the study design.

#### 2.1.2. Red Clover in Clinical Trials

Red clover (*Trifolium pratense* L.) belongs to the legume family and is often used to relieve symptoms of menopause, high cholesterol, and osteoporosis [78]. Isoflavones: biochanin A, formononetin, and sissotrin, are responsible for its estrogenic activity. In intestines, biochanin A and formononetin are demethylated and metabolized to genistein and daidzein [79]. The bone-preserving effects of red clover have also been examined, but not as extensively as those of soy [80].

In a randomized, placebo-controlled study, an isoflavone preparation (Rimostil^®^) containing genistein, daidzein, formononetin and biochanin A was administered to 46 postmenopausal women in a double-blind protocol after a single-blind placebo phase, and followed by a single-blind washout phase. A 6-month-long administration of an isoflavone combination extracted from red clover (57 mg/day or 85.5 mg/day) to postmenopausal females increased the BMD of radius and ulna [44]. In another clinical trial (*n* = 205), the red clover extract containing 41 mg isoflavone per tablet (Promensil^®^) ameliorated the decrease of bone mineral content (BMC) and BMD in lumbar spine in pre-, peri-, and postmenopausal women taking the supplement for 12 months. Authors also reported the elevation of bone formation markers [45].

In another 12-month, double-blind, parallel design RCT, 78 postmenopausal osteopenic women were supplemented with calcium (1200 mg/day), magnesium (550 mg/day), calcitriol (25 mg/day) and given either red clover extracts rich in isoflavone aglycones and probiotics (RCE, 60 mg isoflavone aglycones/day and probiotics) or a masked placebo. RCE intake combined with supplementation (calcium, magnesium, and calcitriol) was more effective than supplementation alone. Twice daily RCE intake over one year prevented a menopause-associated decrease of BMD normalizing bone turnover, promoting a favourable oestrogen metabolite profile (2-OH:16α-OH), and stimulating equal production in postmenopausal women with osteopenia [46]. 

Thorup et al. found that the intake of 150 mL red clover extract containing 37.1mg isoflavones for 12 weeks improved bone health in menopausal women (*n* = 60). The conclusions were based on BMD and T-score at the lumbar spine and plasma CTX levels [47]. However, a review of the potential skeletal benefit of red clover concluded that there was limited evidence of efficacy [81]. For example, in a placebo-controlled 3-year trial in 401 women with a family history of breast cancer, 40 mg of red clover produced no effect on BMD [48].

In another study with perimenopausal women (*n* = 250), when taking two tablets per day containing red clover extract (28.6 mg or 41 mg isoflavones) for 12 weeks, no significant differences in bone turnover markers were observed compared to placebo [49].

Although the evidence is limited, it appears that red clover isoflavones may have a beneficial effect on bone mineral density in peri- and postmenopausal women [80].

#### 2.1.3. Soybean and Red Clover in Animal Studies and In Vitro Models

The studies that investigated the effects of soybean on markers of bone turnover in ovariectomized rats reported contradictory results. Park et al. reported that soybean increased serum osteocalcin levels and decreased urinary deoxypyridinoline (DPD) levels [82], while Byun et al. observed a decrease in osteocalcin and DPD levels [83]. However, other authors detected no influence of soybean on bone turnover markers in ovariectomized rats [84]. Hinton et al. reported that soybean improved whole bone and tissue level biomechanical properties in ovariectomized rats, although it did not improve the trabecular microarchitecture [84].

Soybean proteins contain a high level of phytate, which decreases calcium bioavailability [85], therefore, the investigations assessing the phytate-removed soybean proteins of bone metabolism were conducted. Phytate-removed and deamidated soybean β-conglycinin enhanced calcium absorption from the intestines in ovariectomized rats [85]. As a consequence, an increase in serum calcium level normalized PTH secretion. Suppression of ovariectomy-induced changes in bone turnover was also observed. Additionally, Akao et al. reported a reduction of bone resorption, enhanced BMD, and strengthened bone in ovariectomized rats receiving phytate-removed and deamidated soybean β-conglycinin [85]. However, the influence on trabecular BMD was less prominent than the influence of cortical BMD.

Soy isoflavones bind to ERβ that are expressed in the calcaneus but not in cortical bone [7]. This fact explains why they mainly influence the trabecular bone. In vivo, soy isoflavones through Smad’s activation in osteoblasts lead to the upregulation of the expression of Runx2 and OSX that are important transcription factors involved in osteoblast differentiation and proliferation [86]. Soy isoflavones decreased RANKL levels. They increase the expression of OPG, β-catenin, and Wnt 3a and 7b in osteoblasts. Noh et al. reported that the combination of soy isoflavone and hop prenylflavones (Soy-Hop) had a protective influence on bone in ovariectomized rats [87]. In their study, Soy–Hop administration in a dose-dependent manner reduced ovariectomy-induced elevation of osteocalcin, alkaline phosphatase (ALP), and CTX levels. It also attenuated the ovariectomy-induced expression of RANKL messenger ribonucleic acid (mRNA). A micro-computed tomography (mCT) examination revealed reduced porosity and decreased separation between trabeculae in the femoral epiphysis in Sop-Hop receiving ovariectomized rats [87]. Kim et al. reported that dry-fermented soybean food ameliorated senile osteoporosis in the senescence-accelerated mouse prone 6 (SAMP6) model [88].

In vitro studies demonstrated that daidzein and genistein bound to RANKL within the side residues involved in RANK binding [89] prevented the formation of the complex of RANKL-RANK that activates bone resorption. Additionally, it was demonstrated that soy isoflavones increased Runx2 expression mineralisation in human osteosarcoma Saos-2 cell culture that activated osteoblasts and led to the acceleration of matrix mineralization [89]. Genistein was also shown to be able to elevate ALP activity and decrease RANKL/OPG ratio in Saos-2 [90]. There are data matching the activation of osteoblasts by genistein with its binding to oestrogen receptor β present on osteoblastic cells [91,92]. Genistein is twenty times more selective for oestrogen receptor β (ERβ) than α [93]. Animal studies confirmed that genistein combined with silicon and zinc significantly reduced RANKL expression and prevented ovariectomy-induced bone resorption [94,95]

Daidzein is the most widely studied soy phytoestrogen. Daidzein was also reported to stimulate osteoblast differentiation. It stimulates osteoblasts through the BMP-2/Smad/Runx2 pathway [96]. It was reported that oestrogen receptor signalling, mitogen-activated protein kinase/extracellular signal-regulated kinases (MAPK/ERK), and phosphoinositide-3-kinase/serine-threonine protein kinase B (PI3K/AKT) were involved in osteoblast activation via daidzein [97].

**Figure 3:**
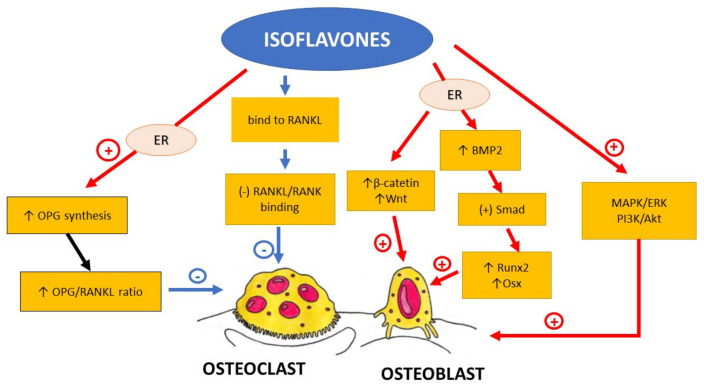
Schematic diagram representing the influence of isoflavones on proliferation, differentiation and activity of osteoclasts and osteoblasts. BMP2—bone morphogenic protein 2, ER—oestrogen receptor, MAPK/ERK—mitogen-activated protein kinase/extracellular signal-regulated kinases signalling pathway, OPG—osteoprotegerin, Osx—Osteoblast-specific transcription factor Osterix, PI3K/Akt- phosphoinositide-3-kinase/serine-threonine kinase signalling pathway, —Receptor Activator for Nuclear Factor κB, RANKL—Receptor Activator for Nuclear Factor κB Ligand, Runx2—Runt-related transcription factor 2, ↑—increased, ↓—decreased, (-)—inhibited, (+)—activated

The summary of the influence of isoflavones on bone metabolism is presented in Figure 3.

#### 2.1.4. Other Plants Containing Isoflavones

##### Alfalfa

Alfalfa (*Medicago sativa* L.), also called lucerne, belongs, as red clover, to the legume family. It is cultivated as a forage crop in many countries over the world. Its sprouts are a common ingredient of dishes made in Indian cuisine. Alfalfa, as other legumes, is a known source of phytoestrogens, including spinasterol, coumestrol, coumestan, and ipriflavone. As mentioned above, the meta-analysis of 63 controlled trials investigating 6427 postmenopausal women revealed that ipriflavone (600 mg/day) is a promising molecule for the prevention and treatment of postmenopausal osteoporosis [15]. Ipriflavone has been reported to induce osteoblast proliferation and prevent menopause-related bone loss.

##### *Pueraria* *candollei var. mirifica*

*Pueraria candollei var. mirifica* (Airy Shaw and Suvat.) Niyomdham (commonly termed *P. mirifica*), known also as *kudzu* root, has a long history as a postmenopausal rejuvenate therapy for indigenous people. It contains various isoflavones: puerarin, daidzein, daidzin, mirficin and salvianololic acid. In a double-blind RCT of healthy postmenopausal women aged 45 to 60 years old, Manonai et al. showed that *Pueraria mirifica* at a dose of 20, 30, and 50 mg/day for a 24-week period demonstrated an oestrogen-like effect on bone turnover. The P1NP level was reduced as seen with other treatments with Erα agonists [50]. In another double-blind RCT, nineteen postmenopausal women (12/7 test/control) received *P. mirifica* powder or placebo for 2 months. Investigators also found a reduced ALP and commented on its relationship to bone preservation, but the isoform targeted was not stated [51].

*Pueraria* extract prevented ovariectomy-induced bone loss in rats [98]. Puerarin, that is, the main active ingredient of *Pueraria* extract, slows down the bone loss and reverses the ovariectomy-induced increase in bone turnover in rats [99]. It also alleviated osteopenia and prevented the deterioration of trabecular structure in mCT [99]. Other authors reported that puerarin inhibited RANKL-dependent osteoclastogenesis [100], induced mineral nodulus formation in osteoblasts through the activation of PI3K/AKT signalling pathway [101] and promotes osteoblast differentiation [102].

Summing up, isoflavones not only prevent bone resorption by the inhibition of RANKL/RANK interaction and osteoclast maturation and differentiation, but they also seem to promote bone formation. They increase, among others, the expression of BMP2 and Runx2, that are involved in the activation and differentiation of osteoblasts. The molecular mechanism of phytoestrogen influence on bone metabolism is very complex and there are many possible pathways that might be involved (Figure 3).

### 2.2. Other Plants Containing Phytoestrogens Investigated in Osteoporosis Treatment

#### 2.2.1. Epimedium (Berberidaceae)

##### Epimedium in Clinical Trials

Epimedium is a genus of about 52 species in the family *Berberidaceae*, which is also known as Rowdy Lamb Herb, Xianlinpi, Barrenwort, Bishop’s Hat, Fairy Wings, Horny Goat Weed, and Yangheye or Yin Yang Huo). The traditional Chinese medicinal herb Epimedii has been utilized for centuries to treat bone fractures, bone loss, and menopause-associated disorders [64]. The results of recent clinical trials have reported suggest that compounds or extracts of Epimedium may prevent or delay the onset of osteoporosis and reduce the risk of hip fractures [21]. Icariin is a prenylated flavonol glycoside isolated from Epimedium herbs, and has been shown to be the main bioactive component [16]. In clinics, Epimedium is used to treat osteoporosis, climacteric period syndrome, breast lumps, hyperpiesia, and coronary heart disease [103].

In a 24-month double-blind RCT in healthy, late postmenopausal women, the intervention group (*n* = 50, a daily dose of 60 mg icariin, 15 mg daidzein, and 3 mg genistein) had a significantly reduced bone loss compared to the placebo group (*n* = 50). Treatment with icariin maintained BMD at 12 months. A long-term (up to 12–24 months) administration of icariin improved BMD in the lumbar spine and femoral neck in a time-dependent manner. Although the effect of icariin is less effective in the improvement in BMD than oestrogen replacement or treatment with bisphosphonates, it seems to be an attractive alternative therapy due to its low risk of severe side effects. It exerted no oestrogenic effect on the uterus and did not change the serum estradiol level, proving its safety when it comes to the endometrium. A 2-year-long treatment with icariin was also not associated with the incidence of breast cancer or cardiovascular events [52]. Further clinical trials encompassing a larger population are needed to investigate the influence of icariin and its derivatives on bone formation and regeneration in humans, as well as its safety profile [16].

##### Epimedium in Animal Models and In Vitro Studies

*Epimedium* flavonoids (icariin, epimedin B, and epimedin C), that possess oestrogenic activity, have been identified as the main constituents of Epimedium plants that exert antiosteoporotic activity, as they inhibit bone resorption, promote bone formation and block urinary calcium excretion [21]. The flavonoids from *Epimedium* promote osteoblast activity through the regulation of the expression of IL-6 (interleukin 6), OPG, RANKL, M-CSF, BMP-2, and Smad4. They modulate the BMP/Smad4 and Wnt/β-catenin signalling pathways, inducing osteoblast differentiation [104]. Icariin is the most abundant flavonoid in *Herba Epimedii* and has a better antiresorptive effect than other components isolated from *Epimedium* plants. It stimulates bone formation by the promotion of osteoblasts differentiation and the enhancement of their activity [16,105]. Icariin activates BMP-2/Smad4, Wnt, and IGF-1 signal transduction pathways [5,17], induces ERK (extracellular signal-regulated kinase), JNK (c-Jun N terminal kinase) and p38 kinase activation [18]. Icariin not only promotes bone formation, but also inhibits osteoclast differentiation and bone resorption. It decreases RANKL-induced osteoclastogenesis via the modulation of NFκB and MAPK expression and downregulation of main regulators of osteoclastogenesis (c-fos and NFAT-c1—nuclear factor of activated T-cells, cytoplasmic 1) [19]. Micro-CT results suggest that icariin improves the bone parameters (BMD, bone volume/tissue volume—BV/TV, connectivity density—Conn.D) and restores bone structure in ovariectomized animals [106]. Ikarisoside A, a flavonoid isolated from *Epimedium koreanum*, also inhibits RANKL-induced osteoclastogenesis [104].

#### 2.2.2. Hop (*Humulus lupulus* L.)

Hop (*Humulus lupulus* L.), which belongs to the Cannabaceae family, has been used worldwide in the brewing industry as a source of bitterness in beer. Apart from this, hop extract is known for containing phytoestrogen components and exerting oestrogenic effects. In general, compounds of the oestrogenically active fraction of lupulin gland secretion belong in the following prenylflavonoids: xanthohumol, being the most abundant prenylflavonoid in hops, izoxanthohumol, 6-prenylnaringenin and 8-prenylnaringenin [107]. Moreover, 8-prenylnaringenin has stronger oestrogenic properties than soy phytoestrogens [108]. Ban et al. reported that hop extract Lifenol^®^ prevented osteoporosis development in ovariectomized rats [109]. Hop extract ameliorated the ovariectomy-induced decreased of BMD, femur weight, and BMC (bone mineral content). Additionally, it restored the trabecular structure of calcaneus bone and inhibited ovariectomy-induced osteoclast activation. A mild osteoprotective effect of hop extract was also reported by other authors [110]. Li et al. reported that xanthohumol blocks RANKL-induced osteoblast differentiation and bone resorption, in vitro and in vivo, in ovariectomized mice [111]. At the molecular level, it blocks the RANKL/TRAF6 (tumour necrosis factor receptor associated factor 6) signalling pathway involved in osteoclastogenesis. Additionally, xanthohumol stimulates osteogenic marker gene expression in mesenchymal and pre-osteoblastic cells [112]. Furthermore, 8-prenylnaringenin, that is, the strongest phytoestrogen known, similarly to soy phytoestrogen, exerts its osteoprotective effect through ERs. It inhibits RANKL expression and induces the expression of ostoprotegerin (OPG), which is an inhibitor of osteoclast activity [113].

## 3. Other Botanicals

### 3.1. Dried Plums

#### 3.1.1. Dried Plums in Clinical Trials

In a rat model of ovariectomy-induced osteoporosis, dried plum (*Prunus domestica* L.) prevented the bone loss and structural damage of bone tissue [114]. The studies described below have tried to confirm this effect in humans, mainly in osteopenic postmenopausal women.

Three-month RCT comparing the influence of dried plums versus dried apples on biomarkers of bone formation in 58 postmenopausal women has revealed that the consumption of 100 g/day dried plums significantly increased the serum markers of bone formation: total ALP, bone-specific ALP (BALP) and IGF-1 [53]. Another one-year RCT compared the effects of daily consumption of 100 g dried plum to 75 g dried apple (control) on BMD and biomarkers of bone turnover in 160 osteopenic postmenopausal women. Hooshamnd et al. reported that dried plum improved lumbar and ulnar BMD when compared to dried apples [115]. Additionally, the authors reported that dried plum increased RANKL and OPG concentration, and decreased serum sclerostin level, however the reported results did not reach statistical significance [54].

Similarly, inconsistent results were obtained in non-randomized six-month intervention trials evaluating the effects of resistance training with and without dried plum at a dose of 90 g in 23 postmenopausal breast cancer survivors. In both groups, an improvement of upper and lower body strength was found, but no improvements in body composition or BMD was detected [116]. However, in a subsequent six-month clinical trial evaluating the efficacy of two doses of dried plum (50 g vs. 100 g) in 48 older postmenopausal women, it was reported that dried plums prevented the loss of total body BMD and reduced the serum concentration of tartrate-resistant acid phosphatase 5b (TRAP-5b). Additionally, the authors concluded that both doses of dried plaums are equally effective [55]. The beneficial effect was also observed in the trial, with 35 men between the ages of 55 and 80 with moderate bone loss. Patients were randomized into one of three groups: 100 g dried plum daily, 50 g dried plum daily, or control group. All three groups also consumed a multivitamin containing 450 mg calcium and 800 IU vitamin D. After three months, decreased serum concentration of osteocalcin was observed, as well as an elevation of OPG/RANKL. Authors suggested that regular consumption of either 100 g or 50 g dried plum for three months may make some contributions to bone formation and bone turnover activity, and a minimal contribution to decreasing inflammation and improving bone density and quality [56].

The results of the presented studies suggest that dried plum is a promising functional food therapy for preventing bone loss, with the potential for long-lasting bone protective effects [114].

#### 3.1.2. Dried Plums in Animal Studies and In Vitro Models

Dried plums contain carbohydrates, vitamins A, B and K, potassium, calcium, magnesium, boron, selenium, dietary fibres, and polyphenols, including chlorogenic acid, rutin and proanthocyanidin [117]. Animal studies comparing the influence of dried plums and standard diet on bone metabolism and bone mechanical properties showed that diet supplementation with dried plums increased vertebral and femoral bone mineral density [118,119]. In ovariectomized animals, the administration of dried plums increased bone mineral density and the number of trabeculae (Tb.N.), and decreased the separation of trabeculae (Tb.Sp.) [120,121]. Further animal studies revealed that polyphenols are the main bioactive compounds responsible for bone response to therapy with dried plums. However, the addition of potassium and vitamin K to the polyphenolic resulted in the additional increase of bone mineral density [122]. In in vitro studies, dried plum polyphenols suppressed osteoclast activity and differentiation [123], increased mineral nodule formation and osteoblast activity [124].

### 3.2. Horsetail (Equisetum arvense)

Horsetail (*Equisetum arvense* L.) is widely distributed over the northern hemisphere. Extracts and other preparations of horsteil have been used for ages in European folk medicine. It contains abundant constituents that may exert beneficial effects on bone health, e.g., silica, flavonoids, and triterpenoids.

The only clinical study evaluating the effectiveness of horsetail in the treatment of perimenopausal osteoporosis recruited 122 women in menopause for at least two years, who had not undergone oestrogen replacement therapy or drug therapy for recalcification: 30 patients were administered with titrated dry horsetail extract for 80 days; 31 patients were administered with placebo for 40 days and titrated horsetail extract for a further 40 days; 29 patients received no treatment whatsoever; 32 patients were treated with Osteosil Calcium for 80 days. All patients received two tablets per day according to procedures for randomized double-blind studies. Patients who received treatment with titrated horsetail extract after the period of placebo administration showed the same changes observed in patients treated with the active ingredient; treatment with titrated horsetail extract and with Osteosil Calcium improved bone metabolism and BMD [57].

*E. arvense* has a high concentration of silica, and it has been demonstrated in vitro that the horsetail extract induced bone regeneration [125] and inhibited osteoclastogenesis [126]. It has been reported that horsetail extract enhanced bone mineralization and bone formation in ovariectomized rats [127]. Additionally, a diet containing horsetail extract (120 mg/kg) increased bone mineral density in rats [128]. However, there are scarce studies to support the hypothesis of the beneficial effects of horsetail on bone health, and the European Food Safety Authority concluded that there is not enough evidence of the bone-protecting influence of *E. arvense* [129].

### 3.3. Black Cohosh (Cimcifuga racemosa)

Data from the following clinical trials suggest the beneficial effects of *Cimicifuga racemosa* on bone metabolism and bone mineral density. Additionally, the authors hint at the possible reduction of the cumulative dose of HRT for the prophylaxis of osteoporosis in patients receiving CR [130].

A double-blind RCT on postmenopausal women showed that CR stimulated osteoblast activity, and improved markers of bone turnover [58]. Other authors reported that *C. racemosa* extract reduced bone resorption (decrease in the urinary level of N-telopeptide) and increased bone formation (elevation of ALP) in postmenopausal women. However, serum obtained from treated females did not stimulated osteoblasts’ culture, but failed to demonstrate a direct stimulating effect of the serum from treated women on a culture of osteoblasts [59]. On the other hand, other authors did not find a bone-favourable effect of *C. racemosa* extract in exercising early postmenopausal women [60]. The absence of a *C. racemosa* -taking non-exercising comparison group was a significant shortcoming of this study, as the possibly positive effect of *C. racemosa* might have been lost in the well-known considerable favourable effect of exercise on BMD [131]. Another trial measuring serum osteocalcin and C-terminal telopeptide [132] did not reveal any significant difference between black cohosh and placebo in measured outcome at 12 weeks.

In animal studies, *Cimicifuga racemosa* increased BMD and restored bone architecture (preventing the decline in BV/TV, Tb.Th., and Tb.N., and preserving SMI—Structural Model Index) in ovariectomized animals [133]. Cycloartane, a tripentoid glycoside isolated from black cohosh, inhibits NFκB and ERK signalling pathways that leads to inhibition of RANKL-induced osteoclast differentiation [134]. Additionally, actein and deoxyactein protect osteoblasts against oxidative stress and promote cell growth and matrix mineralisation [135,136].

### 3.4. Salvia miltiorrhiza and Salvia plebia

Red sage (*Salvia miltiorrhiza Bunge*), also known as Danshen in Chinese, has been used to treat bone diseases in traditional Chinese medicine. Guo et al. analyzed clinical trials that investigated the efficacy of *Salvia miltiorrhiza* in the treatment of osteoporosis. In reported trials *S. miltiorrhiza* was given as monotherapy or as a part of combined therapy with other plants or ingredients. They identified 36 trials that demonstrated high efficacy and no toxicity of *S. miltiorrhiza*, however, in some studies, small patient samples, short treatment duration, frequent lack of detailed numerical data, and no clear endpoints limited their value and reliability [137]. *S. miltiorrhiza* influence on bone regeneration was also investigated in patients with avascular and ischemic necrosis of femoral head. *S. miltiorrhiza* was injected and implantated in the calcium phosphate cement/*S. miltiorrhiza* drug delivery system by minimal invasive surgery. The digital substruction arterography and X-ray films demonstrated that *S. miltiorrhiza* administration improved the microcirculation and regeneration of the affected bone [138].

In animal studies, *S. miltiorrhiza* and *S. plebia* prevented ovariectomy-induced decrease in trabecular bone mass and BMD. It also reduced TRAP5b activity and oxidative stress in ovariectomised animals [22,26,139]. Tashinones, salvianolic acid, and eudebeiolide B have been identified as osteoprotective components of Salivia plants. Tanshinones inhibit the formation of TRAP5b-expressing osteoclasts by suppressing the RANKL-induced expression of c-fos and NFATc1 [22,23]. Salvianolic acid A and B modulate osteoblast differentiation and upregulate osteoblast activity [24,25]. Liu et al. reported that *Radix salviae* improves bone microarchitecture and biomechanical properties through the Wnt/β-catening signalling pathway in ovariectomized rats [140].

### 3.5. Other Herbs

#### 3.5.1. *Labisia pumila* and *Eurycoma longifolia*

*Labisia pumila* that belongs to the family *Myrsinaceae* is used in Asia for the treatment of painful menstruation and disorders of sexual life in females due to its oestrogenic properties. As a phytoestrogen-containing plant, it is also used in osteoporosis treatment [141].

Both *L. pumila* and *E. longifolia* have demonstrated a protective effect on bone loss due to osteoporosis in previously published studies. In a double-blind, 24-week RCT, 119 healthy women (aged 41–55 years) experiencing peri-menopausal or menopausal symptoms were enrolled and supplemented with herbal formulation (Nu-femme™) comprising LP (SLP+^®^) and *Eurycoma longifolia* (Physta^®^) or placebo. There were no significant differences between- and within-group of bone markers for osteoporosis reflecting bone formation (BALP) and resorption (N-terminal telopeptide—NTX) [61].

#### 3.5.2. *Drynaria* *fortunei*

*Rhizoma Drynariae*, the dried rhizome of *Drynaria fortunei (Kunze) J. Sm.*, is reported to prevent age-associated bone loss. It contains mainly flavonoids, triterpenoids, phenolic acids, and glycosides [142]. In ovariectomized animals, *Rhizoma Drynariae* extract prevented oestrogen deficiency-induced weight gain without an unfavourable effect on the uterus [143]. Additionally, it exerted a protective effect on bone, increasing Tb.N. and bone fraction (BV/TV), and decreased Tb.Sp. in calcaneus bone. In vitro studies revealed that *Rhizoma Drynariae* extract inhibits RANK activity [143]. Sun et al. reported that polysaccharides from *Rhizoma Drynariae* exerts an antiosteoporotic effect in ovariectomized rats. It maintained trabecular microarchitecture and bone biomechanical properties, and increased femoral and tibial bone mineral density (BMD) [144].

#### 3.5.3. Other Plant-Derived Constituents

As there is a great need to develop new drugs that might be used in the treatment of osteoporosis, there are plenty of reports on studies investigating the influence of plant-derived bioactive substances on the activity and differentiation of osteoclasts and osteoblasts. Loureirin B (flavonoid, extracted from *Dracaena cochinchinensis*) and kirenol (diterpenoid extracted from the Chinese herbal medicine *Siegesbeckiae*) inhibit RANKL-induced osteoclast differentiation by attenuation of NFAT expression [145,146]. Kaempferol (natural flavonol found in various plants, e.g., tea and broccoli) that exerts oestrogenic properties, on the one hand, inhibits bone resorption and on the other promotes bone formation [147]. Its bone-protective effect is mediated through regulation of oestrogen receptor, bone morphogenetic protein-2 (BMP-2), NF-κB, MAPK and mammalian target of rapamycin (mTOR) signalling pathways [148]. Mangiferin (xanthone originally extracted from mango tree) attenuates ovariectomy-induced osteoporosis in rats [149] and promotes osteoblast differentiation through the increased expression of Runx2 and BMP2/Smad1 signalling pathway [150,151]. Quercetin (flavanol wildly distributed in plants, e.g., red onion) inhibits RANKL-mediated osteoblastogenesis through Wnt, NFκB, Nrf2 (nuclear factor erythroid 2-related factor 2), and SMAD-dependent signalling pathways [152].

## 4. Conclusions

**Figure 4:**
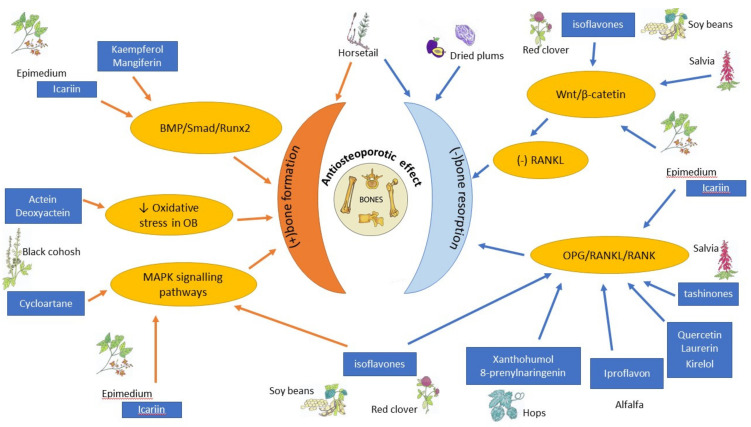
Schematic diagram of the antiosteoporotic activity of various plants and their components. BMP—bone morphogenic protein, MAPK—mitogen-activated protein kinase, OB—osteoblast, OPG—osteoprotegerin, RANK—Receptor Activator for Nuclear Factor κB, RANKL—Receptor Activator for Nuclear Factor κB Ligand, Runx2—Runt-related transcription factor 2, ↓ decreased, (-)—inhibited, (+) activated

Traditional folk medicine is a rich source of bioactive compounds waiting for discovery and investigation that might be used in treatment and prophylaxis od osteoporosis. The mechanism of action of some chosen botanicals are presented in Figure 4 and Table 2.

## Figures and Tables

**Table 1:** Herbal compounds with antiosteoporotic properties investigated in vitro and in animal models.

Herbal Compounds	Subgroup	Chemical Structure	Proposed Mechanism of Action
Daidzein	isoflavones	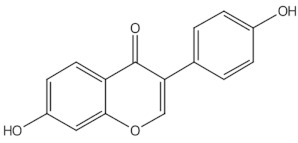	ER mediated signalling pathway, activation of intracellular pathways: AKT, phospholipase C (PLC), mitogen-activated protein kinase (MAPK) [14]
Genistein	isoflavones	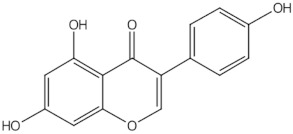	ER-mediated signalling pathway, activation of intracellular pathways: AKT, PLC, MAPK [14]
Ipriflavone	isoflavones	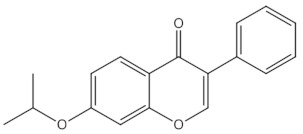	Modulation of key signalling pathways to regulate bone resorption (e.g., ↓urinary DPD, NTX) and bone formation (e.g., ↑BALP and osteocalcin [15]
Biochanin A	O-methylated isoflavones	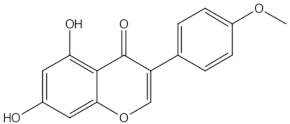	ER mediated signalling pathway, activation of intracellular pathways: AKT, PLC, MAPK [14]
Formononetin	O-methylated isoflavones	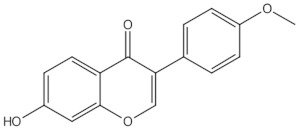	ER mediated signalling pathway, activation of intracellular pathways: AKT, PLC, MAPK [14]
Glycitein	O-methylated isoflavones	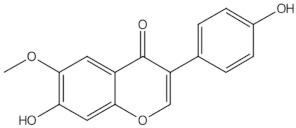	ER mediated signalling pathway, activation of intracellular pathways: AKT, PLC, MAPK [14]
Icariin	prenylated flavonol glycoside	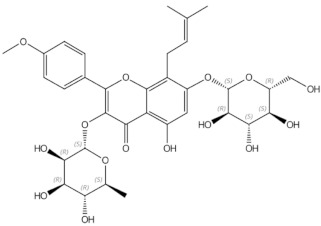	Stimulation of bone formation by promotion of osteoblasts differentiation and enhancement of their activity [16]; activation of BMP-2/Smad4, Wnt and IGF-1 signal transduction pathways [5,17], induction of ERK, JNK and p38 kinase activation [18]; decreasing of RANKL-induced osteoclastogenesis via inhibition of NFκB and MAPK expression [19]
8-prenylnaringenin	prenylflavonoids	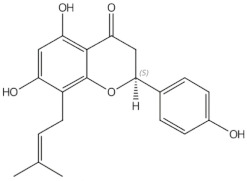	Promotion of osteoblast differentiation and induction of osteoclast apoptosis [20]
Epimedin B	prenylflavonoids	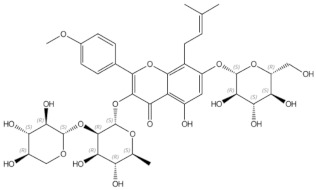	Inhibition of bone resorption, bone formation promotion and urinary calcium excretion blocking [21]
Epimedin C	prenylflavonoids	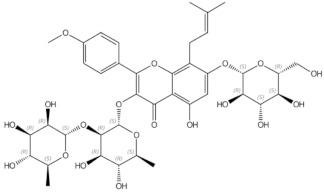	Inhibition of bone resorption, bone formation promotion and urinary calcium excretion blocking [21]
Tanshinones (dihydrotanshinone, tanshinone I, or tanshinone IIA)	diterpenes	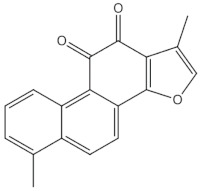 Tanshinon 1	Inhibition of the TRAP5b-expressing osteoclasts formation by suppressing RANKL-induced expression of c-fos and NFATc1 [22,23]
Salvianolic acid A	phenolic acids	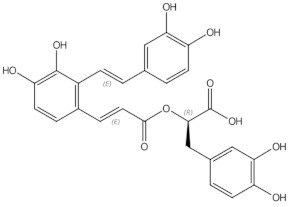	osteoblast differentiation modulation and osteoblast activity upregulation [24,25]
Salvianolic acid B	phenolic acids	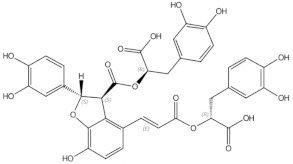	osteoblast differentiation modulation and osteoblast activity upregulation [24,25]
Eudebeiolide B	eudesmane-type sesquiterpenoid	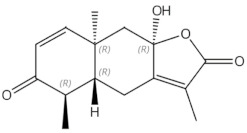	Osteoclastogenesis inhibition and ovariectomy-induced bone loss prevention by regulating RANKL-Induced NF-κB, c-Fos and Calcium Signaling [26]

AKT—protein kinase B, BALP—bone-specific alkaline phosphatase, CTX—type I collagen cross-linked C-telopeptide, DPD—deoxypyridinoline, ER—oestrogen receptor, ERK—extracellular signal-regulated kinase, JNK—c-Jun N-terminal kinase, MAPK—mitogen-activated protein kinase, NFκB—nuclear factor-kappa B, NTX—type I collagen cross-linked N-telopeptide, PLC—phospholipase C, RANKL—Receptor Activator for Nuclear Factor κB Ligand, TRAP 5b—Tartrate-resistant acid phosphatase 5b.

**Table 2:** Summary of potential anti-osteoporotic properties of botanicals in clinical trials.

Botanicals	Population and Design	Intervention	Outcome	Authors and References
Soy isoflavones	single open-group prospective clinical intervention; 42 postmenopausal women,	three daily servings for 12 consecutive weeks of whole soy foods containing approximately 60 mg/day of isoflavones	↓ NTX, ↑ osteocalcin	Scheiber 2001 [27]
Soy isoflavones	RCT with 3 groups: soy rich diet, HRT, control; 187 healthy asymptomatic postmenopausal women aged 39–60,	approximately 47 mg/day of isoflavones in diet group; duration: 6 moths	↑ bone osteoblastic activity but not as effective as HRT in reducing the postmenopausal turnover, ↑ osteocalcin	Chiechi 2002 [28]
Soy isoflavones	RCT with 3 groups: placebo, mid-dose, and high-dose, in pill form; 203 postmenopausal Chinese women aged 48 to 62,	placebo (daily dose of 0 mg isoflavones + 500 mg calcium, *n* = 67) mid-dose (40 mg isoflavones + 500 mg calcium, *n* = 68) and high-dose (80 mg isoflavones + 500 mg calcium, *n* = 68); duration: 12months	favourable effect on rates of change in BMC at the total hip and trochanter among later postmenopausal women (>4 y), in women with lower body weight (≤median, 55.5 kg), or among women with lower level of calcium intake (≤median, 1095 mg/day)	Chen 2004 [29]
Soy isoflavones	RCT with 3 groups: placebo, mid-dose, and high-dose; 90 Chinese postmenopausal women aged 45–60	placebo (daily dose of 0 mg isoflavones) mid-dose (84 mg) and high dose (126 mg), 30 subjects/group; duration: 6months	Retardation of lumbar and femoral bone loss at the lumbar spine (L1–L4) and bone resorption	Ye 2006 [30]
Soy isoflavones	double-blind RCT with 2 groups: placebo, isoflavone conjugates in capsule form, 68 postmenopausal Japanese women	Isoflavone group (75 mg of isoflavone conjugates/day), 34 subjects/group; duration: 12 months	↑ serum equol in the equol producers but not in the nonproducers, preventive effects of isoflavones on hip BMD	Wu 2007 [31]
Soy isoflavones	double-blind RCT with 3 groups: placebo, mid-dose, and high-dose in tablet form; 255 postmenopausal women aged 46–63	placebo (daily dose of 0 mg isoflavones) mid-dose (80 mg) and high dose (120 mg); duration: 3 years	mild beneficial femoral BMD—and SSI	Shedd-Wise 2011 [32]
Soy isoflavones	double-blind RCT with 2 groups: placebo, isoflavones in tablet form; 87 Korean postmenopausal women aged 45–60	Isoflavone group = 70 mg in 2 tablet per day (8.0 mg glycitin, 20 mg daidzein, and 12.4 mg genistin); duration: 12 weeks	↑ serum BALP and osteocalcin	Lee 2017 [33]
Soy isoflavones	RCT with 3 groups; placebo, HRT, phytoestrogens; 325 postmenopausal women	HRT group (1 mg oestradiol and 0.5 mg norethisterone acetate p.o. daily, phytoestrogens group (40% standardized extract with 20 mg soy isoflavones (genistein and daidzein), two capsules = 40 mg p.o. daily; duration: 12 months	no significant differences between the effectiveness of the HRT and phytoestrogen in terms of effects on BMD and bone resorption	Tit 2018 [34]
Soy isoflavones	double-blind RCT with 3 groups: placebo, soy protein, soy protein + isoflavone in snack bar; 200 women within 2 years of the onset of their menopause	placebo (isoflavone of less than 300 parts per billion) PI (15 g soy protein with 66 mg of isoflavones), SP (15 g soy protein alone, isoflavone free) daily, 100 women/group; duration: 6 months	↓CTX with SPI supplementation compared to SP, ↓ P1NP with SPI supplementation	Sathyapalan 2017 [35]
Soy isoflavones	double-blind RCT with 2 groups: placebo, isoflavones in form of tablet	placebo (0 mg of isoflavones), isoflavones extracted from soy protein (200 mg daily = 4tablets) 248 multi-ethnic menopausal women aged 45 to 60; duration: 2 years	not superior to placebo in preventing bone loss or in reducing bone turnover or menopausal symptoms in women in the first 5 years of menopause	Levis 2011 [36]
Soy isoflavones	double-blind RCT with 2 groups: placebo, phytoestrogens; 202 postmenopausal women aged 60–75	placebo (milk protein), phytoestrogens (25.6 g soy protein containing 52 mg genistein, 41 mg daidzein and 6 mg glycetein (aglycone weights; duration: 12 months	no significant differences for BALP, calcium, and phosphorus measurements.	Kreijkamp-Kaspers 2004 [37]
Soy isoflavones	double-blind, multicentre RCT with 2 groups: isoflavone-enriched biscuits and bars and control biscuits and bars; 237 early postmenopausal women aged 53 ± 3y	placebo group (biscuits and cereal bar), isoflavone- enriched foods (soy isoflavone concentrate containing 40–50% of isoflavones) providing a mean daily intake of 110 mg isoflavone aglycones/day; duration: 12 months	isoflavone-enriched products did not alter lumbar and total body BMD or markers of bone formation and bone resorption	Brink 2008 [38]
Genistein	double-blind RCT with 2 groups: placebo, genistein; 389 postmenopausal women	placebo group (calcium and vitamin D, *n* = 191), genistein aglycone group (54 mg/day + calcium and vitamin D, *n* = 198) duration: 36 months	↑lumbar and femoral BMD, ↓bone resorption markers (DPD, CTX, RANKL), ↑ bone formation markers (BALP, IGF-1 and OPG)	Marini 2007 [39]; Marini 2008 [40]
Genistein	double-blind RCT with 2 groups: placebo, genistein; 138 postmenopausal women (age 49–67 years)	placebo (0mg of isoflavones, *n* = 67), genistein (54 mg/day, *n* = 71), duration: 24 months	↑ femoral and lumbar BMD, improvement of the quantitative ultrasound parameters (stiffness index, amplitude-dependent speed of sound, and bone transmission time)	Atteritano 2009 [41]
Genistein	double-blind RCT with 2 groups: placebo, geniVida™ bone blend group; 70 postmenopausal women	placebo (calcium only, *n* = 28), genistein group = 30 mg/daygenistein + vitamin D3 (800 IU/days) + vitamin K1 (150 μg/days) + polyunsaturated fatty acids (1 g polyunsaturated fatty acids as ethyl ester: eicosapentaenoic acid/docosahexaenoic acid ratio = ~2/1, *n* = 30); duration: 6 months	↑ BMD, ↑ BALP and NTX	Lappe 2013 [42]
Genistein	double-blind RCT with 2 groups: placebo, genistein, 121 postmenopausal women	placebo (1000 mg of calcium and 800 IU vitamin D3; *n* = 59), genistein aglycone group (54 mg/day + calcium, vitamin D3; *n* = 62, duration: 24 months	↑ femoral and lumbar BMD, ↑ BALP	Arcoraci 2017 [43]
Red clover isoflavones (genistein, daidzein, formononetin, biochanin A)	double-blind RCT with 4 groups: placebo, red clover isoflavone preparation (Rimostil) in 3 doses, 46 postmenopausal women	placebo, Rimostil (phytoestrogens)—28.5 mg, 57 mg, or 85.5mg/day, duration: 6 months,	↑ BMD after 57 mg and 85.5 mg/day	Clifton-Bligh 2001 [44]
Red clover isoflavones	double-blind RCT with 2 groups: placebo, isoflavone supplement Promensil^®^; 205 pre-, peri-, and postmenopausal women aged 49–65	placebo, isoflavone supplement (providing 26 mg biochanin A, 16 mg formononetin, 1 mg genistein, 0.5 mg daidzein/daily); duration: 12 months	↑ bone formation markers (BALP, P1NP), ↓ lumbar spine BMC and BMD	Akinson 2004 [45]
Red clover isoflavones	double-blind, parallel RCT with 2 groups: placebo, red clover extract; 78 postmenopausal osteopenic women supplemented with calcium 1200 mg/day, magnesium 550 mg/day, calcitriol 25 µg/day	placebo, red clover extract (60 mg isoflavone aglycones/day + probiotics); duration: 12 months	↓ lumbar and femoral BMD loss, ↓ CTX	Lambert 2017 [46]
Red clover isoflavones	double-blind RCT with 2 groups: placebo, red clover extract; 60 menopausal women	placebo, red clover extract (daily dose of 150 mL containing 37.1 mg isoflavones = 33.8 mg as aglycones); duration: 12 weeks	↑ spinal BMD	Thorup 2015 [47]
Red clover isoflavones	double-blind RCT with 2 groups: placebo, standardized red clover isoflavone dietary supplement (Promensil^®^); 401 healthy women aged 35–70 years	Placebo, red clover isoflavones (40 mg/day); duration: 36 months	safe and well tolerated but no effect on BMD	Powles 2008 [48]
Red clover isoflavones	double-blind RCT with 3 groups: placebo and 2 dietary supplements derived from red clover, 252 menopausal women ages 45–60 years	placebo, Promensil^®^ (82 mg total isoflavones), Rimostil^®^ (57.2 mg total isoflavones), duration: 12 weeks	no effect on bone turnover markers.	Knudson Schult 2004 [49]
Kudzu root (*Pueraria candollei var. mirifica*)	double-blind RCT with 4 groups: placebo, 3 dose of Pueraria; 71 postmenopausal women aged 45 to 60 years	placebo (*n* = 20), Pueraria mirifica in capsules (20, 30, or 50 mg once daily, *n* = 51); duration: 24 weeks	↓ BALP	Manonai 2008 [50]
Kudzu root (*Pueraria candollei* var. *mirifica*)	double-blind RCT with 2 groups 19 postmenopausal women	placebo tablet, tablet containing 25 mg dried PM root powder, 4 tablets/day; duration: 2 months	↓ ALP	Okamura 2008 [51]
Epimedium	double-blind RCT with 2 groups: placebo, Epimedium-derived phytoestrogen flavonoids (EPF), 100 healthy late postmenopausal women	placebo (*n* = 50), EPF group (*n* = 50; a daily dose of 60 mg Icariin, 15 mg daidzein, and 3 mg genistein), +300 mg calcium daily for both group; duration: 24 months	↑ lumbar and femoral BMD, ↓ DPD,	Zang 2007 [52]
Dried plums	RCT with 2 groups: placebo (dried apples), dried plums; 58 postmenopausal women	placebo (dried apples 75 g daily), dried plums (100 g daily); duration: 3 months	↑IGF-1, ↑ ALP, ↑ BALP	Ajamandi 2002 [53]
Dried plums	RCT with 2 groups: placebo, dried plums, 160 postmenopausal women with osteopenia	placebo (dried apples 75 g daily), dried plums (100 g daily) + 500 mg Calcium, 400 IU (10 μg) vitamin D daily for both group; duration: 12 months	↑ ulnar and lumbar BMD, ↓ BALP	Hooshmand 2011 [54]
Dried plums	RCT with 3 groups: placebo, 2 dose of dried plums, 48 older postmenopausal women	control (0 g/day dried plum), dried plum (50 or 100 g/day dried plum), duration: 6 months	↑ BMD, ↓ TRAP-5b, ↑ BALP/TRAP-5b ratio	Hooshmand 2016 [55]
Dried plums	RCT with 3 groups: placebo, 2 dose of dried plums; 35 men between the ages of 55 and 80 with moderate bone loss	control group (0g prunes), 100 g prunes daily, 50 g prunes daily, + multivitamin containing 450 mg calcium and 800 IU vitamin D for all group, duration: 3 months	↓ osteocalcin, ↑ OPG/RANKL ratio	Ajmandi 2020 [56]
Horsetail (*Equisetum arvense*)	Double blind RCT with 4 groups: control, placebo + horsetail extract, horsetail extract, calcium, 122 women in menopause for at least two years	no treatment/control group (*n* = 29), placebo for 40 days and titrated horsetail extract for a further 40 days (*n* = 31), titrated dry horsetail extract for 80 days (*n* = 30); Calcium (Osteosil^®^) for 80 days (*n* = 32), After the 80-day initial study period, patients treated with titrated horsetail extract and with calcium continued treatment for one year	↑ in the average densitometric values for the vertebra	Corletto 1999 [57]
Black cohosh (*Cimcifuga racemosa*)	double-blind RCT with 3 groups: placebo, black cohosh, oestrogens; 62 postmenopausal women	placebo, black cohosh (40 mg of herbal drug/day), conjugated oestrogens (0.6 mg/day); duration: 12 weeks.	↑ osteoblast activity, weak estrogen-like activity, no significant effects on coagulation markers and liver enzymes	Wuttke 2006 [58]
Black cohosh (*Cimcifuga racemosa*)	prospective clinical trial with 2 groups: untreated control, isopropanolic extract of *Cimicifuga racemosa*, 82 postmenopausal women	control group (*n* = 37), isopropanolic extract of *Cimicifuga racemosa* (Remifemin^®^, 40 mg/day, *n* = 45), duration: 3 months	↓NTX (marker of bone resorption), ↑ ALP (marker of bone formation)	Garcia-Pérez 2009 [59]
Black cohosh (*Cimcifuga racemosa*)	RCT with 3 groups: control (CG), exercise group (EG), exercise and *Cimicifuga racemosa* (CR) supplementation group (EGCR), 128 early postmenopausal women	CG (wellness control, *n* = 42), EG (*n* = 43), EGCR (40 mg/day of CR BNO 1055; *n* = 43), Calcium (1500 mg/d) + vitamin D (500 IE/d) supplementation for all participant duration:12 months	CR (CR BNO 1055) did not enhance positive effects of exercise on BMD at the lumbar spine	Bebenek 2010 [60]
*Labisia pumila* and *Eurycoma longifolia*	double-blind RCT with 2 groups: placebo, Nu-femme™, 119 healthy women aged 41–55 years experiencing peri-menopausal or menopausal symptoms	placebo (*n* = 59), herbal formulation (Nu-femme™, *n* = 60) = 200mg Labisia pumila (SLP+^®^) + 50mg Eurycoma longifolia (Physta^®^); duration: 24 weeks	No significant effect on bone formation (BALP) and resorption (NTX) markers	Chinnappan 2020 [61]

ALP—alkaline phosphatase, BALP—bone-specific alkaline phosphatase, BMC—bone mineral content, BMD—bone mineral density, CTX—type I collagen crosslinked beta C-telopeptide, DPD—deoxypyridinoline, HRT—hormonal replacement therapy, IGF-1– insulin-like growth factor 1, NTX—type I collagen crosslinked N- telopeptide, OPG—osteoprotegerin, P1NP—type I procollagen-N-propeptide, RANKL—Receptor Activator for Nuclear Factor κB Ligand, SSI—strength strain index, ↑—increased, ↓—decreased

**Table 3:** Four chemical forms of main isoflavones.

Aglycones	Glycosides	Acetylglycosides	Malonyl Isoflavone Glycosides
Daidzein	Daidzin	Acetyldaidzin	Malonyldaidzin
Genistein	Genistin	Acetylgenistin	Malonylgenistin
Glycitein	Glycitin	Acetylglycitin	Malonylglycitin
Biochanin A	Sissostrin		Malonylsissostrin
Formononetin	Ononin		Malonylononin
Daidzein	Daidzin	Acetyldaidzin	Malonyldaidzin

## Data Availability

No new data were created or analyzed in this study. Data sharing is not applicable to this article.

## Abbreviations

AKT	protein kinase B
ALP	alkaline phosphatase
BALP	bone-specific alkaline phosphatase
bFGF	basic fibroblast growth factor
BMC	bone mineral content (BMC)
BMD	bone mineral density
BMP	bone morphogenic protein
BMSC	bone marrow-derived mesenchymal stem cells
BV/TV	bone fraction
c-FMS	colony-stimulating factor-1 receptor
Conn.D.	connectivity density
CTX	type I collagen crosslinked beta C-telopeptide
DPD	deoxypyridinoline
ER	oestrogen receptor
ERK	extracellular signal-regulated kinases
FasL	Fas ligand
bFGF	basic fibroblast growth factor
Hh	Hedgehog
HRT	hormonal replacement therapy
IGF	insulin-like growth factor
IL-6	interleukin 6
JNK	c-Jun N terminal kinase
MAPK	mitogen-activated protein kinase
M-CSF	macrophage colony-stimulating factor
mCT	micro-computed tomography
mRNA	messenger ribonucleic acid
mTOR	mechanistic target of rapamycin
NFAT-c1	Nuclear factor of activated T-cells, cytoplasmic 1
NFκB	nuclear factor-kappa B
Nrf2	nuclear factor erythroid 2-related factor 2
NTX	type I collagen crosslinked N- telopeptide
OB	osteoblast
OC	osteoclast
OPG	osteoprotegerin
OSX	Osterix
P1NP	type I procollagen-N-propeptide
PDGF	platelet-derived growth factor
PI3K/AKT	phosphoinositide-3-kinase/serine-threonine protein kinase B
PLC	phospholipase C
pre-OB	pre-osteoblasts
pre-OC	pre-osteoclasts
PTH	parathyroid hormone
RANK	Receptor Activator for Nuclear Factor κB
RANKL	Receptor Activator for Nuclear Factor κB Ligand
RCT	randomized clinical trial
Runx2	Runt-related transcription factor 2
SERM	Selective Oestrogen Receptor Modulator
sFasL	soluble Fas lignad
SMI	Structural Model Inde
Tb.N.	number of trabeculae
Tb.Sp.	separation of trabeculae
Tb.Th.	trabecular thickness
TGFβ	tumour growth factor β
TRAF6	tumour necrosis factor receptor associated factor 6
TRAP 5b	Tartrate-resistant acid phosphatase 5b
